# Family Attitudes regarding Newborn Screening for Krabbe Disease: Results from a Survey of Leukodystrophy Registries

**DOI:** 10.3390/ijns6030066

**Published:** 2020-08-20

**Authors:** Karlita Blackwell, Michael H. Gelb, Anna Grantham, Natasha Spencer, Christin Webb, Tara West

**Affiliations:** 1 Parent Advocate & Consumer Representative, Saint Louis, MO 63021, USA; karlitab23@gmail.com; 2 Department of Chemistry, University of Washington, Seattle, WA 98195, USA; gelb@uw.edu; 3 Department of Biochemistry, University of Washington, Seattle, WA 98195, USA; 4 Hunter’s Hope Foundation, P.O. Box 643, Orchard Park, NY 14127, USA; 5 Parent Advocate & Consumer Representative, Chicago, IL 60649, USA; spencernatasha@hotmail.com; 6 Parent Advocate & Consumer Representative, Powell, TN 37849, USA; webbc@clintonschools.org; 7 Duke University PBMT and Cellular Therapy Program, Durham, NC 27705, USA; tara.west@duke.edu

**Keywords:** newborn screening, lysosomal storage diseases, Krabbe disease, leukodystrophy, Globoid cell leukodystrophy

## Abstract

Newborn screening (NBS) for Krabbe disease (KD) is currently underway in eight states in the USA, and there is continued discussion of whether to implement KD NBS in additional states. Workgroup members sought to survey a large number of families affected by KD. Families in KD and leukodystrophy family registries were contacted to seek their participation in *The Krabbe Newborn Screening—Family Perspective Survey*. The 170 respondents are comprised of the following: 138 family members with a KD individual diagnosed after development of symptoms, 20 notified about KD via NBS, and 12 with a KD individual diagnosed through family history of KD. The key results are that all NBS families with an early-infantile KD family member elected to pursue hematopoietic stem cell transplantation therapy. Of the 170 responders, 165 supported the implementation of KD NBS in all states in the USA.

## 1. Introduction

Newborn screening (NBS) is warranted when there exists a valuable treatment for a rare disease and when the window of opportunity for effective treatment is lost if patients are diagnosed only after the development of irreversible symptoms. Additional criteria include: (1) an understanding of the natural history of the disease; (2) a high-throughput newborn screening test that gives a minimal number of false positives and negatives; and (3) an ability to establish a post-NBS follow-up plan.

NBS for a leukodystrophy known as Krabbe disease (KD) started in New York state in 2006, soon after it was shown that early cord blood transplantation favorably alters the natural history of the disease [1,2,3]. NBS for KD was added to additional state panels after New York (Illinois, Indiana, Kentucky, Louisiana, Missouri, New Jersey, New Mexico, Ohio, Pennsylvania, South Carolina, Tennessee), and KD NBS has started in Indiana, Kentucky, Missouri, New Jersey, New York, Ohio, Illinois and Tennessee. Patient advocacy played a large role in the addition of KD NBS to these state panels. The requirements for adding a new condition to NBS panels differ in each state.

The expansion of NBS panels in the USA is also driven by a majority vote from the Advisory Committee on Heritable Disorders in Newborns and Children, and gains final support from the Secretary of Health and Human Services. After a condition is vetted by this group and approved by the Secretary, it becomes part of the federal Recommended Uniform Screening Panel (RUSP). States try to include all conditions on the RUSP on their individual panels. In 2010, by an eight-to-seven vote, KD was not recommended for addition to the RUSP. The Committee asked for additional information about the algorithm for NBS of early infantile KD, as well as more information about the specific benefits of treatment.

In the past few years, the precision of NBS for KD has increased. All NBS laboratories screening for KD first measure the activity of the relevant enzyme galactocereborisdase (GALC) in dried blood spots (DBS), because this has a sufficiently high throughput for NBS. As mentioned, New York was the first state to start NBS for Krabbe disease. Over the past ~13 years, New York has found ~30–50 newborns per year exhibiting GALC activity below their cutoff. DBS from these newborns are submitted for GALC DNA sequence analysis. However, such analysis is often inconclusive because of the widespread occurrence of DNA variants of unknown significance and the difficulty in integrating variants that partially reduce the activity of GALC [4]. More recent work has shown that measurement of the lipid biomarker psychosine greatly increases the precision of KD NBS [5]. Disease-specific reference ranges of psychosine have now been established for infantile KD and for late-onset KD [5]. Furthermore, psychosine measurements can be made using the same dried blood spot used for GALC analysis and can thus be incorporated into NBS. With GALC and psychosine measurements carried out prior to issuing the NBS report, the number of families in New York facing infantile or high-risk late-onset KD is estimated to be <3–5 per year. New York has recently started to include psychosine analysis in DBS as part of KD NBS. Kentucky started NBS for Krabbe disease about three years ago. GALC enzymatic activity is measured, along with five other lysosomal enzymes. Post-analysis bio-statistical tools (Collaborative Laboratory Integrated Reports, CLIR) are used to eliminate many of the false positives. Psychosine analysis on the same DBS is the final step in the process. To date, Kentucky has reported no false positives or false negatives for KD NBS, and a single newborn with early infantile KD [6]. Missouri checks low-GALC samples for the 30-kb deletion in the *GALC* gene (pathogenic for KD) and carries out DNA sequencing as well if the GALC activity is at the low end of the range below their cutoff. Missouri has just started to incorporate psychosine analysis into their NBS method. Illinois now obtains psychosine for all below-cutoff-GALC newborns, as well as employing GALC DNA sequencing. Ohio only measures GALC in DBS, and newborns with below-cutoff values are referred to a regional genetics center. New Jersey obtains DNA sequencing for GALC below-cutoff newborns, and is planning on adding psychosine as part of its NBS in the next ~1 year. KD NBS data has been published only for New York and Kentucky. All states now screening for KD NBS either incorporate psychosine measurement as part of NBS or are taking steps to include psychosine testing in their algorithm. Thus, a lack of precision in KD NBS is no longer a concern going forward.

The first five early infantile KD NBS-positive patients in New York state had several factors leading to a delay in treatment, which negatively impacted patient outcomes [4]. Long-term follow-up of KD patients identified via family history who were treated by hematopoietic stem cell transplantation have been published [1,7,8]. Furthermore, new data on treated patients identified through NBS will be reported in the near future, which show improved outcomes since the first eight years in New York. These newer cases reflect the changes made in Krabbe disease NBS and follow-up and are similar to patients identified early through family history.

Long-term follow-ups of KD patients identified via family history who were treated by hematopoietic stem cell transplantation have been published [1,7,8]. Data on treated patients identified through NBS will be reported in the near future and show a similar outcome to patients identified early through family history.

Here we summarize the findings of a widespread survey of families affected by KD NBS. This includes families with a KD-affected family member diagnosed through NBS or independently of NBS, as well as families touched by NBS who have a currently asymptomatic family member.

## 2. Method

*The Krabbe Newborn Screening—Family Perspective Survey* was created by the authors to better understand the perspective of families affected by KD. A copy of the survey is provided in the Supplementary Materials. The workgroup used Google Forms to create and disseminate the survey, which was open for 2 months (12 December 2019 to 22 February 2020).

A contact list of 439 individuals from the Hunter’s Hope Foundation registry was generated from families affected by KD, as well as families whose child had a positive screening for the disease. In addition, Hunter’s Hope Foundation publicized the survey on the foundation’s Facebook (12,954 followers), Instagram (3073 followers), and Twitter (3380 followers) pages. Parent authors (K. Blackwell, N. Spencer, and C. Webb) distributed the KD survey through social media platforms, sharing on personal pages; group pages designed to promote KD awareness to the general public (806 and 18,958 members); in a private, international, group for families affected specifically by KD (793 members); as well as private, international groups for those affected by leukodystrophy as a whole (2666 and 1467 members). Surveys were also distributed via personal email accounts. Hunter’s Hope Foundation sent email blasts to the aforementioned contact list on 12 December 2019 and again on 25 January 2020.

## 3. Results

### 3.1. Survey Method

Survey questions included basic biographical information. Additionally, families were asked if their KD-affected family member was identified by diagnosis after the development of symptoms (symptomatic diagnosis, SD), whether they had family history of the disease (family history diagnosis, FHD), or whether they were contacted by their state NBS laboratory (NBS). Survey results were organized based on these three groups of families. The survey also included questions about eligibility of the patient for treatment, whether they received treatment, whether they advocated for KD NBS in their state, and whether they are supportive of NBS for KD throughout the USA. The survey also allowed participants to provide comments about their answers to the previous question and an opportunity to share any additional comments.

### 3.2. Survey Results

Table 1 summarizes the number of survey responses, the number of families in each group (SD, NBS, FHD), the relationship of the responder to the KD-affected patient, the location of residence at the time of the patients’ birth, and the type of KD the patient was diagnosed with (age of onset).

**Figure 1:**
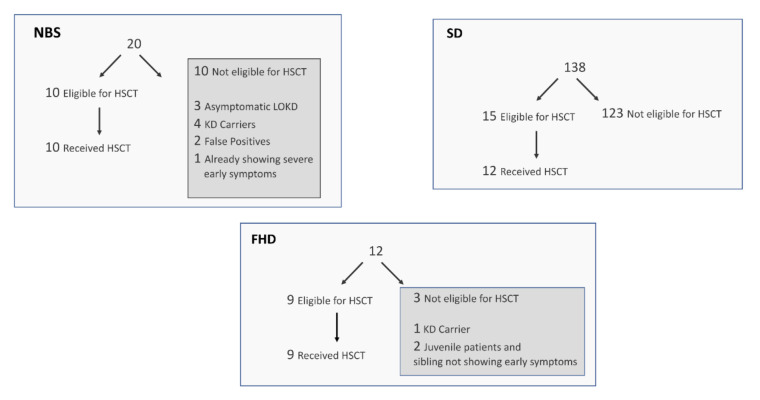
Eligibility of patients to receive a transplant and whether families opted for this treatment for the NBS, SD, and FHD groups (see text for discussion). HSCT—hematopoietic stem cell transplantation; LOKD—late-onset Krabbe disease.

Figure 1 summarizes the KD patients in terms of eligibility to receive a transplant and whether families opted for this treatment. For the NBS group, 10 of 20 were eligible for transplant, and all 10 elected to have the transplant. Among the 10 not eligible for transplant, five newborns were identified as KD carriers, three were identified as asymptomatic newborns with a risk of developing later-onset KD, one was already symptomatic with early infantile KD, and two were identified as false positives for NBS of KD. Among the FHD group, three of 12 were not yet eligible for a transplant. Of the three ineligible patients, one is a carrier of the disease and is not at risk for developing symptoms, and the other two have juvenile onset KD, so a transplant would not be accessible until just prior to the predicted onset of disease. Nine of 12 FHD families were eligible for a transplant, and all of these families elected to have the transplant. Among the SD group, 15 of 138 were eligible for a transplant. Three of these 15 refused treatment, because their child was already symptomatic and transplant would likely not reverse the demyelination that already occurred.

The percentages of each group who advocated for KD NBS in either the state their child was born in or currently resides in were: SD (62% of 138), NBS (30% of 20), and FHD (58% of 12). The percentages of each group who believed that KD should be included on every state’s NBS panel were: SD (98% of 138), NBS (90% of 20), and FHD (100% of 12).

In the final two questions of the survey, we first asked if families supported the expansion of KD NBS throughout the USA, and then asked families to comment on their previous answers. All comments are provided as Supplementary Materials. A summary of the responses is given here. All nine NBS KD families receiving a diagnosis of early-infantile KD opted for treatment by hematopoietic stem cell transplantation. Most of the families indicated that NBS for KD allowed them the choice to treat their child and that treatment offered their child a chance at life. The five families who found out that their child was a carrier for KD all considered the process life-saving and viewed the results as information to protect future generations. Among the three families who found out that their child was at risk of developing later-onset KD but was asymptomatic at the time of the survey, two supported NBS for KD and were grateful for the knowledge and the opportunity to save their child’s life if the disease did present symptoms. The other had a negative experience with the post-screen follow-up and wished they had never found out. Of the two families who were told that their child was a false positive for KD, one supported NBS for KD and believed every state should protect newborns from the deadly disease. The other had a negative experience with their immediate follow-up and found the lack of protocol and information from their medical providers traumatizing. It should be noted that the two NBS KD families that were unsupportive of nationwide NBS for KD both cited gross ambiguity in the information given by their medical providers throughout the process as the reason for their position. These events illustrate the need for improvement of some aspects of the KD NBS process. The other 10 families in the NBS group were supportive of nationwide KD NBS in the USA.

All 12 families in the FHD group supported nationwide KD NBS in the USA. They all commented that early treatment would be possible with NBS, which would save lives.

Among the 98% of families in the SD group who supported nationwide KD NBS in the USA, most of the comments show that these families believed: (1) KD NBS will be lifesaving for babies born with KD through treatment intervention prior to the onset of symptoms; (2) it is the right of the parents/caregivers (not medical providers or legislators) to make an informed decision as to whether or not their child will benefit from available treatment; (3) KD NBS is the only way to save future babies from what their children have already suffered through; (4) cost should not be an issue when it comes to saving a life; (5) a family’s zip code should not be the determining factor as to whether or not a child is diagnosed in time for eligibility to receive available treatment. Among the three SD families who did not support nationwide KD NBS in the USA, two said they did not support KD NBS unless it provided a normal life/cure for the child and one family member said KD NBS is expensive and is not possible in every state.

## 4. Discussion

As stated throughout this chapter, the survey was focused on families affected by KD and not medical professionals who diagnose and treat KD patients. Thus, the survey covers a very small sector of the population and survey results may be different if it were carried out on the general population. Through the platform of social media, families now participate in a disease community that the medical professionals and legislators should listen to and learn from. Affected families have a way of viewing the defeats and successes of each family category represented in this survey, including more than twenty years of children who received transplants. The leukodystrophy community is a well-informed group, working in daily collaboration with one another to improve their children’s quality of life. Their experiential knowledge and insight are sought-after by medical providers and researchers and are intrinsic to understanding the natural history of the disease to continue to advance KD treatment. Families want the next generation of affected families to benefit fully from the treatment options that are available. Of all survey participants, 58% have politically advocated for NBS for KD and a total of 97% feel that KD NBS should be implemented in every state.

Going forward, there are two new components of KD NBS that are worth noting. In the past, false positives were obviously a concern, as described in the introduction. Early on in the New York state KD NBS program, it was hypothesized that DNA sequencing of the gene relevant to KD (*GALC*) would eliminate most false positives, but interpretation of DNA sequences has been straightforward only in some cases, and inconclusive in others, due to the presence of variants of unknown significance and because of the combination of GALC variants that can partially reduce GALC activity. Measurement of residual GALC enzymatic activity in leukocytes is helpful but also inconclusive in some cases. The lipid biomarker psychosine has been discovered to be highly diagnostic and prognostic for KD [5]. To date, all early-infantile KD patients show psychosine levels in DBS above 10 nM, and newborns with 2–10 nm psychosine are considered at high risk to develop late-onset KD [5]. Kentucky was the first NBS lab to measure psychosine in DBS as a second-tier test when the initial GALC enzyme levels were low. Psychosine analysis is done as part of NBS, i.e., before families are notified of a positive screening result. Kentucky has reported no false positives for KD after ~4 years of NBS [6]. Most states now screening for KD NBS have recently added psychosine analysis as part of NBS, and the remainder are planning to incorporate this biomarker analysis in the near future. With psychosine as part of KD NBS, the false positive rate is expected to be very low, and similar or below the false positive rates found in other conditions that are part of state NBS panels.

Notably, every category of parents in this survey noted dissatisfaction with the information, resources, and medical care/follow-up protocols available after receiving a positive screen or diagnosis. This is consistent with previous research on parents’ attitudes toward NBS, dating as far back as 2003, which documents the correlation between parents’ negative opinions of NBS and the lack of adequate information from their medical providers. Additional parallels are found in analysis of the way in which well-informed medical providers support the mental well-being of parents in similar circumstances [9]. With the majority of surveyed families supporting NBS-KD and opting for HSCT when possible, it is clear that parents are willing to take on the risks associated with the treatment. It is important to consider the severity of KD if left untreated. In addition to the extreme suffering of the child, an untimely death is the outcome for 100% of these patients.

KD is a very rare disease, and thus not all genetic disease specialists are fully prepared to deal with the follow-up procedure that comes after NBS. Recently, the Krabbe NBS Council has been created to provide guidance to all physicians of children identified by KD NBS. This group is specifically focused on improving follow-up protocols and information provided to patients at risk of developing later-onset KD (beyond the early-infantile period) but who are currently asymptomatic. The Council is composed of the top KD treatment teams in the world, members of the New York KD NBS consortium that started when New York first began implementing KD NBS, at least one member of the NBS laboratory from each state that now screens for KD, and the researchers who developed NBS for KD based on GALC enzymatic and psychosine measurement. The Krabbe NBS Council is in the process of preparing a publication that provides expert consensus for the management of children who are at risk of developing late-onset KD, as well as an update to the earlier guidelines chapter on early-onset KD [10]. With the new KD NBS guidelines, including psychosine measurement as part of NBS, it is anticipated that all newborns with early KD will be identified, as well as <3–4 newborns in each year per state in the USA who are at a significant risk of early- or late-onset KD.

## Figures and Tables

**Table 1:** Summary of survey responses from 170 responders. KD—Krabbe disease; SD—symptomatic diagnosis; NBS—newborn screening; FHD—family history diagnosis.

Number of Responders	Responder Groups	Relationship of Responder to KD Individual	Location of Residence	KD Onset
170	138 (81%) SD group	124 (73%) mothers	135 (79%) USA	125 (88%) early-infantile (0–6 months
	20 (12%) NBS group	17 (10%) fathers	14 (8%) Europe	27 (19%) late-infantile (0.5–3 years)
	12 (7%) FHD group	29 (17%) non-parents	9 (5%) S. Africa	4 (2.8%) juvenile (3–8 years)
			7 (4%) Canada	2 (1.4%) adult
			5 (3%) Africa,	4 (2.8%) uncertain
			Brazil, Australia	

## Supplementary Materials

Supplementary materials can be found at https://www.mdpi.com/2409-515X/6/3/66/s1.
