# Supplementary material for: Family Attitudes regarding Newborn Screening for Krabbe Disease: Results from a Survey of Leukodystrophy Registries
Source: Int J Neonatal Screen. 2020 Aug 20;6(3):66. doi: 10.3390/ijns6030066 (PMC7570074; doi:10.3390/ijns6030066)
Supplement: Supplementary file 1 [file IJNS-06-00066-s001.zip › IJNS-831718-supplementary.pdf]

Supplemental Material

Item 1. *The Krabbe Newborn Screening - Family Perspective Survey*

Item 2. All family comments

Krabbe Newborn Screening - Family Perspective Survey

This anonymous survey is for parents and/or individuals affected by Krabbe Disease via a positive Newborn Screen, or symptomatic diagnosis with the disease. Your participation in this survey will be used in a patient perspective article for a medical journal to help providers understand affected families' experience and perspective. If you have more than one child affected by Krabbe Disease please complete the survey for each child.

* Required

Relationship to the individual affected by Krabbe Disease: *

- ☐ Self
- ☐ Mother
- ☐ Father
- ☐ Legal Guardian
- ☐ Grandparent
- ☐ Sibling
- ☐ Other:

If other, please specify:

Your answer

In what year was your child born? *

Your answer

If deceased, please include the year of their death:

Your answer

What country was your child born in? *

Your answer

If in the United States, what city and state were they born in? *

Your answer

How did you learn your child may be affected by Krabbe Disease? *

- Through my state's newborn screening program
- At (or before birth) due to having an older sibling affected by Krabbe Disease
- Due to symptoms of the disease (not through newborn screening or carrier screening).
- Other:

If your child has been diagnosed with Krabbe disease, what type does your child have? *

Choose

If your child has been found to test positive for Krabbe disease by the state newborn screening laboratory, what did the follow-up professionals tell you?

Choose

From your answer to the above question, how old was your child when you received this information from the follow-up professionals?

Your answer

Was your child eligible to receive a cord blood/bone marrow transplant to alter the effect of the disease? *

- Yes
- No

Did your child receive a cord blood/bone marrow transplant? *

- Yes
- No

Have you advocated for the addition of Krabbe Disease in your state's NBS program (either in the state your child was born or the state in which you currently reside)? *

- Yes
- No

Do you believe newborn screening for Krabbe Disease should be implemented in every state *

- Yes
- No

In your own words, please explain your response to the previous question: *

Your answer

Additional Comments (If you would like to elaborate on any of your answers or feel there are relevant issues that were missed by the survey, please include them here):

Your answer

Relationship to the individual affected by Krabbe Disease: *

- [ ] Self
- [ ] Mother
- [ ] Father
- [ ] Legal Guardian
- [ ] Grandparent
- [ ] Sibling
- [ ] Other:

If other, please specify:

Your answer

In what year was your child born? *

Your answer

If deceased, please include the year of their death:

Your answer

What country was your child born in? *

Your answer

If in the United States, what city and state were they born in? *

Your answer

How did you learn your child may be affected by Krabbe Disease? *

- Through my state's newborn screening program
- At (or before birth) due to having an older sibling affected by Krabbe Disease
- Due to symptoms of the disease (not through newborn screening or carrier screening).
- Other:

If your child has been diagnosed with Krabbe disease, what type does your child have? *

Choose

If your child has been found to test positive for Krabbe disease by the state newborn screening laboratory, what did the follow-up professionals tell you?

Choose

From your answer to the above question, how old was your child when you received this information from the follow-up professionals?

Your answer

Was your child eligible to receive a cord blood/bone marrow transplant to alter the effect of the disease? *

- Yes
- No

Did your child receive a cord blood/bone marrow transplant? *

- Yes
- No

Have you advocated for the addition of Krabbe Disease in your state's NBS program (either in the state your child was born or the state in which you currently reside)? *

- Yes
- No

Do you believe newborn screening for Krabbe Disease should be implemented in every state *

- Yes
- No

In your own words, please explain your response to the previous question: *

Your answer

Additional Comments (If you would like to elaborate on any of your answers or feel there are relevant issues that were missed by the survey, please include them here):

Your answer

Submit

Never submit passwords through Google Forms.

This form was created inside of Hunter's Hope Foundation. Report Abuse

Google Forms

Survey Comments

NBS Group

Awareness	Awareness And early detection is essential.
ID Carriers	Our children were flagged as carriers, which will help them make educated decisions about their own children. If they HAD the disease, they would have been able to receive early treatment because of newborn screening.
ID Carriers	If it wasn't for the screening I would have no idea my daughter is a carrier!
ID Carriers	My daughter is a carrier for Krabbe disease and my family went back 3 generations of it through genetic testing and are just now finding out. I am grateful for the new born screening because now we have the tools and resources for future generations.
No NBS	This test took away a lot of joy in the first 3 Months of my child's/family's life for a false positive. For a disease that currently has no cure, this false positive brought extreme sadness, fear, and wrestling with what to do as many people in our lives had opinions on if we should continue testing or not after receiving the initial positive result. We spent a lot of money to receive uncertain results each time with no definitive answers. It wasn't until a random research program was found by our genetic physician that we got results stating that it was a false positive. Had he not found this, or we ran out of money to continue to test, we would be left wondering if our son would die for the rest of his life. My genetic physician did not have his child tested for only this disease on the newborn screen and we see why. The testing is poor and for something so serious, I'm so incredibly disappointed in how this process occurred. I understand the need for more research to find a cure, but the strain financially, emotionally, and mentally was incredibly painful. There just has to be a better way to do this. Especially when hospitals aren't equipped to handle what to do next when a false positive is detected.
No NBS	We had absolutely no answers to any questions. All they could tell us was they don't know when and how it will affect our daughter. We also have a 4 year old that had to be tested and she came up positive as well. Both girls have no symptoms and are very

	advanced for their age. I honestly wish we never found out. It has done nothing but add stress to our lives.
Other	Newborn screening should be done for Krabbe Disease and serious diseases like it that can be tested for at this age.
Parental Rights/Knowledge	It's very important every mother and child have the opportunity to know head on if their child may be affected , to prevent the heartache
Parental Rights/Knowledge	But also parents need to know all the facts about the children that have survived it also. I remember getting all negative but true facts. I was told that my daughter would not survive with her two mutations and high risk. I had faith and now my daughter is 11 no symptoms and doing great. I did speak to her specialist watching her in Rochester and let her know that I would love to meet with any parents near us to help them get through any questions or etc. I feel the parents need that because there is so much about krabbe on internet and docs that did scare me so much and I was in dark about I did basically become a pro on looking up info etc. I would love to help these parents through the process and maybe being involved in the other side of krabbe and give them the faith and courage to go to the hunter hope page to be able to read the scary stuff but still have hope. I feel I would like to give back some of the good I learned to help others. I would love to work with Jill or her mom who I did meet a few times to be that other piece of the puzzle that helps make hunters hope take off even higher and help awareness

Save/Protect Lives	I believe screening should be done for any test which could save a child's life, by knowing ahead of time what they may be facing. We have done several walks and raised thousands of dollars to raise awareness for this horrific disease. We are lucky my son's levels are low enough not be effected by this disease and we are blessed to do all we can to help others.
Save/Protect Lives	I believe every state should protect newborn babies from this potential deadly disease
Save/Protect Lives	I believe that newborn screening provides each child the chance at a life that would otherwise be taken by a fatal and devastating disease. Not to mention the long and suffering complications from it prior to death. I believe that because of newborn screening, my child is alive, attending preschool and thriving today. Each parent deserves to make the decision that is best for them, their child and their family. And each child deserves the chance at life.
Save/Protect Lives	Krabbe and many leukodystrophies are terrible diseases that cause prolonged suffering for the affected children as well as their families. While Krabbe is rare, when untreated its severe nature is something that no child should ever have to endure. Infants, toddlers, and children should not have to suffer a slow and painful death. Newborn screening saves lives, prevents unnecessary suffering, and provides affected children the chance to experience full and joyful lives.
Save/Protect Lives	My daughter is at risk. Knowing the symptoms and following up with doctors will save her life if the disease ever presents itself.
Save/Protect Lives	We lived in a state that didn’t test when I had my son but he was born in a state that did test. Not because we knew only because we live right on the state line. Everyday I can’t imagine if I would have had my son in Tennessee he would most likely not be with us today.

Treatment Intervention	If detected on time per my understanding there is chance to help relieve symptoms and to make it less painful for a child
Treatment Intervention	Newborn screening in our state led to very early diagnosis which then allowed us to get to Dr. Escolar and her team in Pittsburgh for evaluation and a cord blood transplant which saved our child's life!!!!
Treatment Intervention	The newborn screening detect my son Krabbe disease as soon as 1 week old and he had his first transplant at one month. After two transplant, he can walk and starting to talk like a normal kid. Not all kid will have the same results but if every state had newborn screen, the child and the parents has the option or has better chances of having a normal life.
Treatment Intervention	Our child would be deceased or dying eight now if we weren't given the chance to watch him grow up courtesy of dr escolar and bmt team in Pittsburgh
Treatment Intervention	It should never matter what state or zip code you live in that determines your child's fate! My daughter is proof that the treatment currently available is enough!!

SD Group

No NBS	Not unless, it provides a normal life for the child.
No NBS	There's no cure, so why test? It does not meet the criteria of "having an acceptable treatment protocol in place that changes the outcome for patients diagnosed early with the disease."
No NBS	Today, the screening is very expensive and I believe is not possible apply for all newborn.
Other	Ok

Other	I am unsure of this answer - I need more information regarding the pros and cons of routine newborn screening. I am not informed regarding, cost of screening, false positive rate, current treatment options, treatment outcomes, etc. and if screening would be for all leukodystrophy or just Krabbe.
Other	Yes
Other	We are fighting to add Krabbe to the newborn screening panel in Virginia.
Other	Every state should test for things such as Krabbe and other leukodystrophies
Other	Don't know anything about this
Other	Every country. Would be nice! Wasn't aware of Krabbes, lost a son and a niece shortly after their first birthday.
Other	Debe implementarse en todo el Mundo, esta es una enfermedad maldita. Aunque Gracias a ella Dios me dio un Hermoso hijo que estuvo con nosotros durante 11 Bellos meses. Es la luz que guía mi camino.
Other	Rare doesn't mean unimportant, period.

Other	No baby or parent should have to be born with this disease.
Other	Yes, no matter how much it cost. Our state shot it down in the house . Cause of founding issues.
Other	Some non mortal diseases are tested, why not this one which is particularly awful ??
Other	Our response then was they would wait to see what other states did. There are more screening now, so it may he a better time.
Other	We have fought and it is truly difficult to get state legislatures to mandate NBS for Krabbe. We got a law passed to mandate education about the availability of NBS tests, but that is a very small step. The largest argument is in the testing techniques, and what they consider “false positives” for carrier status vs actual affected status. It is a full time job to try and get our state to care about our children.
Other	NBS for Krabbe Disease should be implemented by every state and province in North America.
Other	My daughter in law was part of the team that advocated for Illinois screening & it was passed in 2018
Other	We have tried twice in NH with no luck

Other	Every child should be screened at birth, it should not depend on what state you live in as to if your child is screened for Krabbe or any disease for that matter!!
Other	I am currently advocating Newborn Screening for Krabbe Disease in NC
Other	There should be an option to check for all diseases, for every child.
Other	No child or family should have to go through what we and our son went through. And are still going through.
Other	It would be great this to added in Greece too. However, I think that never will be done.
Other	When our son was born in 2011, we, as a community, were asking this very question. For the duration of his 7-year, non-transplanted life, we continued to ask it. One-and-half-years after his death, here we are still. Meanwhile, more states have added NBS for Krabbe Leukodystrophy to their panel: seven are currently screening for Krabbe Disease while five more have passed legislation.<br><br>Why stop to contemplate whether newborn screening for Krabbe Disease should be implemented in every state when it's already happening. I realize New York, Missouri, Kentucky, Tennessee, Illinois, New Jersey, Ohio, New Mexico, Pennsylvania, Louisiana, Indiana and South Carolina vs. the remaining thirty-eight states hardly constitutes a tipping point, but this question holds us back, keeps us from valuing the progress we have made.<br><br>When I review the criteria that governs the classic argument on wether or not to screen for an illness, in the case of Krabbe Disease, yes, the cart was put before the horse, and it has proven beneficial. We now use elevated psychosine values as evidence of disease progression before symptoms present. This simple blood draw eliminates the need for an extensive battery of

	tests every six months for those children who have been identified through NBS but are asymptomatic. A new generation of children have been transplanted experiencing gains in quality of life that can now replace the 10-year-old, outdated, data that so frequently gets sited by our critics. We have a published consensus of guidelines for the newborn screening, diagnosis and treatment of Ifantile Krabbe Disease that can "guide public health laboratories on methodologies for screening and inform clinicians about the need to promptly diagnose and treat infantile Krabbe disease."<br><br>On a personal level, as a mother of a deceased child, I will alway have wanted more for my son: a successful transplant, of course, yes, nothing less! But until there is a cure or a prevention, there will always be shortcomings and misgivings. This is human nature. From the standpoint of the larger picture however, we have made significant strides in a short period of time even if the larger medical community keeps telling us we are doing it all wrong!
Other	States that don’t screen, id love to ask each member of the NBS board if it would matter if their child was diagnosed with Krabbe
Other	I testified in front of the Ohio State House to implement it. I've also written term papers for my Bachelor's degree on it.
Other	Barbra Boxer told us to get lost!
Other	so many baby are going threw so much. And it breaks my heart

Parental Rights/Knowledge	Every child should be screened - regardless of history. I was adopted and had no medical history and no way of knowing I was a carrier. It was too late for my daughter when we learned she had the disease. Not to mention that many in the medical field are unfamiliar with this disease, the symptoms and the rapid progression so when diagnosed it is often too late to do anything for the affected child.
Parental Rights/Knowledge	Every parent deserves the chance to fight for their child's life and quality of life. We were robbed of that ability due to the lack of education in the medical community that attempted to diagnose our son. We were going to be referred to the NIH: Undiagnosed Diseases Program but had to run 2 more genetic tests before we could apply. The very last genetic test showed one deletion on my side and from there the doctors decided to run a lysosomal enzyme function test that proved he had too little GALC in his system confirming the diagnosis of Krabbe along with one minor spelling change in my husband's DNA.
Parental Rights/Knowledge	No one should have to go through what we did, especially the emotions of finding out that had it been caught at birth we could have tried to save her life. THAT was the worst part of the entire diagnosis reveal. Not screening for Krabbe at birth robs parents of the right to TRY to save their child's life. That right should rest in the hands of parents, not the government. That's why we fight for improved NBS in our state, and we will continue to support families in other states until every baby has the same chance at a healthy life.
Parental Rights/Knowledge	I believe that all states and other countries should have NBS. No parent should ever have to wonder if they are doing something right. My child has Krabbe and it took us until she was 5 months old to get diagnosed. It's so devastating. We never thought that or imagined our life would be like this at all.
Parental Rights/Knowledge	I believe parents should have the information needed to make an appropriate decision of what would be best for their family/child.
Parental Rights/Knowledge	Newborn screening give families a choice. With the advances in medicine, we as parents deserve to know before it's too late to do anything. We did not have a choice with our son but had he been screened at birth, his outcome could possibly have been different.
Parental Rights/Knowledge	In my way, newborn screning for krabbe disease should be implemented because nowadays, krabbe, is much more commun and it is so letal! Everyone should know if the newborn has or not krabbe disease!

Parental Rights/Knowledge	I never knew the word krabbe until last February when we found out our grandson has it. I don't want any family to lose a child to this horrid disease. I want parents to have options rather than hear the words go home, love him and watch him die.
Parental Rights/Knowledge	Giving multiple children a better quality of life is all I can ask for. No one should go through what I went through, watching and then losing a child with Krabbe. Parents should be given a better choice than what I was given and early detection is the only way to do this. We need to provide knowledge for these families.
Parental Rights/Knowledge	Newborn screening is very very importat for the parents, to give them some control of this terrifyed disease
Parental Rights/Knowledge	Parents shouldn't have to go through 6 or more months of agony wondering what is wrong with their child, what they could have done differently. Or if you are missing something and you are not fixing it soon enough.
Parental Rights/Knowledge	NBS is about giving the families an option before it is too late. The family does not have to transplant but the information is critical if that is the option they choose. Less importance on false positives is necessary. I would rather be scared for a few weeks then miss an opportunity of life saving treatment from no testing at all. My son could be walking if he was born in New York!
Parental Rights/Knowledge	I believe that parents need to have access to any and all newborn screening panels and if they choose not to participate then that is their choice. but I believe it needs to be known for parents that aren't aware that there is other testing that can be done that the state doesn't require.
Parental Rights/Knowledge	No parent should have to go through what my family went through because they did not know the signs and symptoms of Krabbe Disease.
Parental Rights/Knowledge	Not having the chance to be educated until after the fact is not only a twisted misuse of power but also grave injustice by the state legislature.
Parental Rights/Knowledge	It will save so many parents from the pain and agony of losing a child and parents should have a right to know if their newborn has this rare disease or not. The babies should have a chance to live a long life and not suffer in pain.
Parental Rights/Knowledge	Every child and their parents have the right to know if they are carriers and about Krabbe's disease

Parental Rights/Knowledge	You cannot control or manage what you dont know. Rather find out sooner than later.
Parental Rights/Knowledge	Every parent should know at the earliest possible date whether their child has Krabbe to minimise the effect of the disease.
Parental Rights/Knowledge	I never new the true cause of my daughter's illness till 3 days before her death and then they could not tell me the type of leukodystrophy till after special testing on her brain tissue, which came back known as Krabbe disease
Parental Rights/Knowledge	It was very rare and I didn't have any idea about Krabbe, I believe that if my baby girl would have been screened for that she would have live longer
Parental Rights/Knowledge	Every state should test, it's the right of the parents and the baby to know and decide on best treatment plan before it's too late.
Parental Rights/Knowledge	It gives any Krabbe child an opportunity at life because it is invisible at birth and you think you are taking home a healthy baby (especially after screening with other newborn tests)
Parental Rights/Knowledge	This is about families having options. No legislator or scientist's opinion matters - families should have the right to intervene with current treatments if they desire and this should not be decided by anyone else but the parents. For this reason I support newborn screening for Krabbe in all states to preserve this option that I feel is a right to improve the life of their child. Forget about the opinions of scientists and doctors that don't have children with the disease.
Save/Protect Lives	If diagnosed in time you can save lives!
Save/Protect Lives	It could have save my grandson's life.
Save/Protect Lives	We were able to get new born screening passed in Ohio. Every child deserves a chance to live.
Save/Protect Lives	This should be done to protect children from the horrible suffering and lack of information.
Save/Protect Lives	It would have saved my daughters life

Save/Protect Lives	IF POSSIBLE., WHY NOT SAVE EVERY CHILD FROM THIS HORRIBLE DISEASE
Save/Protect Lives	The argument I hear from doctors in our state is that there is unnecessary stress that comes with false positives. I also heard that research isn't far enough to help the children so there is no cure and no hope. State leaders/doctors on the board who make the calls need to be better educated and understand that there is hope and there is treatment and there are options for kids who are tested and diagnosed at birth. It would have given our child a chance. Unnecessary stress is welcomed as a Krabbe parent if it helps save even one life - or extend the quality of life.
Save/Protect Lives	Had we known she had desease we could have taken proper steps to save our daughter's life!
Save/Protect Lives	No child or parent should ever have to endure the pain and suffering Krabbe brings without a chance at life
Save/Protect Lives	Earlier the detection, the better results
Save/Protect Lives	Every child not tested at birth is living with a death sentence. We have the technology, we need to use it. Putting these children (and parents) through the pain of life with Krabbe is inhumane.
Save/Protect Lives	Absolutely, every state should implement newborn screening for Krabbe. Why would you not, if there is a process that can save a child's life?
Save/Protect Lives	While it would not have saved her life, we could have been prepared for when the symptoms started instead of her almost starving and suffering for months before the diagnosis
Save/Protect Lives	Had we known she had desease we could have taken proper steps to save our daughter's life!
Save/Protect Lives	If they tested here in UK we could of had longer time and better quality of life for him

Save/Protect Lives	My daughter would have at least been given a chance at life.
Save/Protect Lives	A poke can save a life and family from devastating circumstances and so much sadness
Save/Protect Lives	A simple blood draw could have saved my son's life. Why wouldn't we test if it could save a life.
Save/Protect Lives	I would like to see more children screened to save lives
Save/Protect Lives	My nephew's life could possibly have been saved through newborn screening or he may have suffered less because his parents would have known he had KRABBE before he started showing symptoms. He suffered for months before they had answers.
Save/Protect Lives	All lives matter.

Save/Protect Lives	My child should have been given a chance at life and not the suffering she went through because she was not screened properly.
Save/Protect Lives	My son's life could have been saved if he was screened at birth. Even if there wasn't a viable treatment option- knowing he had a fatal disease would have saved us so much money from medical testing and lessened his suffering to get him on the correct medication.
Save/Protect Lives	If by implementing a simple blood test that could save many lives then we should fight to have it in every state.
Save/Protect Lives	No child should have to go through anything, like Krabbe, if there is a chance to detect it at birth to help give a chance of life.
Save/Protect Lives	NBS CAN save lives, or at least give our children a better quality of life for a longer period.
Save/Protect Lives	Every baby deserves a Krabbe free life
Save/Protect Lives	I believe every child in the world should be tested at birth, they deserve it. It's all we have until a cure for this terrible disease has been found....
Save/Protect Lives	Krabbe disease is the worst nightmare a family can ever have. Watching your beautiful baby suffer as the disease progresses doesn't come close to the physical pain that they endure. Almost all of the babies diagnosed are given a death sentence because once symptomatic, there is nothing that can be done. We will never be the same again. Our family is broken and lost without our little girl. She is so missed.
Save/Protect Lives	My son would still be alive or atleast had a chance to live.

Save/Protect Lives	My nephew was diagnosed with Krabbe Disease at 6 months old. Knowing that a newborn screening could have saved his life motivates myself and our family to fight for this so that no other family has to go through this devestating experience.
Save/Protect Lives	Newborn Screening should be provided in every state, because these babies deserve the best chance at life!
Save/Protect Lives	This must be done to save newborn babies from developing this disease.
Save/Protect Lives	É muito importante e urgente fazer-se o teste! É a única forma de salvar uma vida! Nada paga a dor e a vida dos nossos filhos!
Save/Protect Lives	Simple: It can save lives!!! Our son did not show symptoms until 3 years old. If Georgia had newborn screening we could have given him a transplant before he showed symptoms.
Save/Protect Lives	I know the transplant isn't an easier option. I know it requires chemo, time spent in the hospital, more testing and monitoring, but the odds of a child still being here and having a better quality life improve with the transplant. I know my child would still be able to see me, smile at me, and be a part of our ordinary everyday lives. He would still be here.<br><br>Even if we didn't want to go through the transplant option, we could have been prepared with the appropriate medications and equipment with the knowledge of his disease at birth. He wouldn't have had to suffer for 3 months as we figured out what was wrong with him and how to treat his symptoms. We wouldn't have had to go to the doctors 4 times and spend a week in the hospital as the ran every type of diagnostic test. We could have been prepared and saved him additional suffering.
Save/Protect Lives	This disease is horrible, and after it settles in there is nothing that can be done. The time is at birth - that is the only time that they have a chance to fight it. We owe it to our children to give them the best chance they can have.

Save/Protect Lives	eVRYCHULD
Save/Protect Lives	No one should suffer with those disease.
Save/Protect Lives	If these diseases were screened for at birth, many lives could be saved. They would suffer less and have far more quality of life.
Save/Protect Lives	If we have the ability to detect for Krabbe at birth, we should be doing that as it can be a lifesaving test that can easily be done with other newborn testing already being done at birth.
Save/Protect Lives	I think it could save lives by screening them
Treatment Intervention	Including krabbe Testing in every newborn screening will help quick intervention for those who may have the disease..
Treatment Intervention	Early detection would have allowed my daughter to receive treatments before the symptoms were too great.
Treatment Intervention	My feelings regarding NBS range from angry and hurting to simply confused. I lost my child to a disease that could have been treated, if only I had known. I feel the country is negligent in not screening for all diseases. They give false hope that a child is being protected (when they screen for only a few diseases and not all). In Arizona 39 of the 66. The excuse of " lack of funding and false positives" makes my blood boil. Quite simply, I find it derelict that the US government does not protect life after birth, when the technologies exsist to do so today.
Treatment Intervention	All family's have an right to choose to be transplanted or not
Treatment Intervention	This should be tested in the newborn screening so that there could be a chance of transplant to prolong life or stop the disease

Treatment Intervention	I would have liked to have been given the choice of a transplant at birth, instead of being told after symptoms presented themselves that there was nothing that could be done.
Treatment Intervention	Krabbe disease isn't as rare as many may think and the progression is devastating. Every state should implement the testing for Krabbe on the newborn screening because there is treatment available if caught at birth. This would at least give families a chance to do everything they can to improve their child's quality of life and even save their life. My daughter was never given that chance and she suffered her whole short 18 month life. My daughter passed away right after the state of IL implemented Krabbe screening. She is a hero in the state of IL, but also nation wide. Please give other kiddos a chance at life. Too many have passed without even being diagnosed and it's just awful.
Treatment Intervention	IF THE NEW BORN SCREENING WILL HELP WITH A CORD BLOOD TRANS PLANT IT CERTAINLY WOULD BE WORTH IT.
Treatment Intervention	The prevention of symptoms and even death could be the result of newborn screening. Our son would still be living.
Treatment Intervention	I would hope that if we had had early screening, we could have taken advantage of transplant services. Our son was too old and they felt too far progressed.
Treatment Intervention	Early intervention is the only medical hope!
Treatment Intervention	It is the only chance to treat/cure.
Treatment Intervention	Every newborn should be tested for any and all screenable and treatable diseases.
Treatment Intervention	Although there is also no cure for Cancer, there are treatments. And like Cancer when diagnosed early, you have choices. That's all I ask for with Krabbe. Both diseases are terminal when not diagnosed early enough and there is no screening at birth to say you'll have Cancer, but there is for Krabbe. Give the parents the opportunity to save their children's lives.
Treatment Intervention	More accurate statistics of people effected with the disease. If detected early treatment can be given

Treatment Intervention	Newborn screening gives our children a chance to receive treatment before becoming symptomatic!
Treatment Intervention	It is important because if the disease is diagnosed immediately it can be transplanted
Treatment Intervention	Newborn screening is the only hope for life-saving treatment.
Treatment Intervention	The earlier it's diagnosed, the sooner treatment can be started
Treatment Intervention	I believe that all children should be tested for krabbe at birth or even while in the mother's womb, if possible, to be prepared for bone marrow transplant as soon as possible. It is possible that bone marrow transplant could even be done on the child while still in utero by removing the fetus, operating, and placing the fetus back into the womb. It has been proven that krabbe begins to effect children while in utero. I find myself asking if there were more anatomy scans/ultra sounds than just one we would have caught the demylenation of my Daughter! I really wanted them to do another and I should have asked for one. But since it wasn't, "normal," protocol I did not. I believed my Baby was going to be perfectly healthy. I don't really understand why more ultrasounds are not necessary during pregnancy. It seems western medicine is very reactive instead of proactive. To save lives we need to be proactive. So now knowing that my Husband and I are carriers there are more options for us and I believe I will be monitored more closely. Why was I not given that option for my first pregnancy. I know numbers show that most children are born perfectly fine but that is not always the case and we as a country need to be prepared to help these innocent babies anyway we can. Needless to say if krabbe would have been on the NBS panel in SC we could have possibly saved my daughter's life. Just saying those words is heart wrenching. I constantly pray for a cure for krabbe and I know that the God I serve is able to

	do exceedingly above all that I could ever ask or think of Him! Leaning on his promises helps us get through this nightmare we call life.
Treatment Intervention	Early diagnosis is critical for a child to receive a blood chord transplant
Treatment Intervention	Krabbe is a devastating disease which causes babies to suffer and die. If this can be avoided and if treatment is available, newborn screening should be done
Treatment Intervention	If NBS was available in Ohio when my son was born he could have been treated prior to onset of symptoms.
Treatment Intervention	I said yes, mainly for being able to catch (hopefully) those late infantile cases. I struggle with wishing I had more options for a longer better life for my child, yet knowing that the likely quality of life my child would have had with a transplant is nothing I would want for him or myself in his place. In the immediate grief phase I would have wanted the transplant.
Treatment Intervention	All disease that are screenable and treatable MUST be tested.

Treatment Intervention	We fight for every kind of disease known to children. Like vaccines and cancer treatments and rare diseases. So why not Krabbe. Why is it that difficult to implement a screening at or before birth to survive a child from a difficult journey?<br>Makes no logical sense.
Treatment Intervention	If we knew about Krabbe my son could have received a cord blood transplant like his younger brother did. It could have saved his life.
Treatment Intervention	So that each child has the opportunity for treatment and a better quality of life
Treatment Intervention	I would want to see a more advanced, kinder treatment than a transplant and I hope research will acheive this. I believe newborn screening should be available to all.
Treatment Intervention	I believe every person with this disease should have the option to have treatment as soon as possible so that they don't have to suffer a horrible death.
Treatment Intervention	To try and treat if possible before symptoms
Treatment Intervention	In South Africa newborns aren't currently screened, resulting in missdiagnosis and untimely deaths and no treatment for those affected by it.
Treatment Intervention	Early detection equals early treatment
Treatment Intervention	Would help with diagnosis of desease
Treatment Intervention	If we had known our child had krabbe disease at birth, he would have been transplanted and most likely have no symptoms.
Treatment Intervention	I wish I had received my child's diagnosis earlier so that she could have been transplanted earlier, had fewer complications, and lived a longer, happier, and less painful life.

Treatment Intervention	It is imperative for early detection , to assure proper treatment.
Treatment Intervention	It's imperative for children to be screened at birth for Krabbe disease so they can have effective treatment.
Treatment Intervention	This can help early detection and quick intervention if transplant is required
Treatment Intervention	Of course! Over the years we've witnessed many children who were successfully transplanted. You can't put a price on extending and/or improving your child's life.
Treatment Intervention	early diagnosis may help - may give opportunity to some. access to medical assistance is also paramount
Parental Rights/Knowledge	It makes it understandable for parents at the begining of infant life

FHD Group

Other	it must be a must!
Save/Protect Lives	Help to save life's
Save/Protect Lives	Yes it can save lives
save/Protect Lives	I am currently advocating Newborn Screening for Krabbe Disease in NC

Save/Protect Lives	Ya to save lives
Treatment Intervention	Yes I do so children can get the proper care as soon as possible
Treatment Intervention	All treatable and screenable diseases should be tested
Treatment Intervention	Every child deserves a chance at life, with or without limitations. Considering there is available testing and treatment, it should be no question. It is inhumane, in my opinion, to allow so many to go undetected and suffer unnecessarily while their parents endure the “diagnostic odyssey.”
Treatment Intervention	It’s important for babies to be tested at birth so they can receive treatments right away.
Treatment Intervention	So that each child has the opportunity for treatment and a better quality of life
Treatment Intervention	Its imperative for children to be diagnosed at birth in order to receive treatment.
Treatment Intervention	A child can be saved by getting a quick diagnosis of Krabbe, so I would support newborn screening if it can return a result within a few days because receiving the cord blood transplant as soon as possible is vital to the child's future prospects.
